# Chinese pediatric *Tuina* on children with acute diarrhea: a randomized sham-controlled trial

**DOI:** 10.1186/s12955-020-01636-1

**Published:** 2021-01-06

**Authors:** Taoying Lu, Lingjia Yin, Ruoqing Chen, Huiyan Zhang, Jianxiong Cai, Meiling Li, Lin Dai, Conghao Zhu, Yongping Zhang, Feng Xiang, Li Wang, Lu Li, Lixin Wang, Darong Wu

**Affiliations:** 1 State Key Laboratory of Dampness Syndrome of Chinese Medicine, The Second Affiliated Hospital of Guangzhou University of Chinese Medicine, PO Box 510120, 111 Dade Road, Guangzhou, China; 2 Program for Outcome Assessment in TCM, The Second Affiliated Hospital of Guangzhou University of Chinese Medicine, PO Box 510120, 111 Dade Road, Guangzhou, China; 3 Health Construction Administration Center, Guangdong Provincial Hospital of Chinese Medicine, Guangzhou, China; 4 Department of Global Public Health, Karolinska Institutet, Stockholm, Sweden; 5 Clinical Epidemiology Division, Department of Medicine Solna, Karolinska Institutet, Stockholm, Sweden; 6 TCM-Integrated Hospital of Southern Medical University, Guangzhou, China; 7 Gastroenterology Department, Guangzhou Hospital of TCM, Guangzhou, China; 8 Acupuncture and Tuina Department, Wenzhou Hospital of Chinese Medicine, Wenzhou, China; 9 Pediatric Department, Dongguan Kanghua Hospital, Dongguan, China; 10 Department of Tuina, Affiliated Hospital of Changchun University of Chinese Medicine, Changchun, China

**Keywords:** Pediatric *Tuina*, Sham *Tuina*, Children, Acute diarrhea

## Abstract

**Background:**

Pediatric *Tuina* has been widely used in children with acute diarrhea in China. However, due to the lack of high-quality clinical evidence, the benefit of *Tuina* as a therapy is not clear. We aimed to assess the effect of pediatric *Tuina* compared with sham *Tuina* as an add-on therapy in addition to usual care for 0–6-year-old children with acute diarrhea.

**Methods:**

Eighty-six participants aged 0–6 years with acute diarrhea were randomized to receive pediatric *Tuina* plus usual care (*n* = 43) or sham *Tuina* plus usual care (*n* = 43). The primary outcomes were days of diarrhea from baseline and times of diarrhea on day 3. Secondary outcomes included a global change rating (GCR) and the number of days when the stool characteristics returned to normal. Adverse events were assessed.

**Results:**

Pediatric *Tuina* was associated with a reduction in times of diarrhea on day 3 compared with sham *Tuina* in both ITT (crude RR, 0.73 [95% CI, 0.59–0.91]) and PP analyses (crude RR, 0.66 [95% CI, 0.53–0.83]). However, the results were not significant when we adjusted for social demographic and clinical characteristics. No significant difference was found between groups in days of diarrhea, global change rating, or number of days when the stool characteristics returned to normal.

**Conclusions:**

In children aged 0–6 years with acute diarrhea, pediatric *Tuina* showed significant effects in terms of reducing times of diarrhea compared with sham *Tuina*. Studies with larger sample sizes and adjusted trial designs are warranted to further evaluate the effect of pediatric *Tuina* therapy.

**Trial registration:**

Clinicaltrials.gov, Identifier: NCT03005821, Data of registration: 2016-12-29.

## Background

Pediatric acute diarrhea is the most common cause of morbidity, mortality and presentation at health services in low- and middle-income countries [1, 2]. Although diarrhea mortality has declined substantially in the past 25 years, diarrhea remains one of the leading causes of death among children younger than 5 years old and has become an important global health problem as well as a heavy burden of disease [1, 3, 4]. In view of the severity of pediatric diarrhea, it is very important to seek effective treatments to reduce morbidity and mortality due to diarrhea.

Although the methods recommended by the World Health Organization (WHO) played an important role in the treatment of diarrhea, some interventions, such as zinc supplementation and oral rehydration solution (ORS), were not easily accepted by children or their parents, which might lead to low compliance and consequently impact the treatments’ effect [5–7]. A survey showed that the coverage and demand of ORS in many developing countries were very low, with less than 40% of children under 5 years old receiving ORS as a treatment for diarrhea [8]. If children with diarrhea are not treated in a timely and appropriate manner, dehydration, electrolyte imbalance, or even death may occur.

Pediatric *Tuina* is one of the therapeutic methods in traditional Chinese medicine (TCM). As it is simple, high performing, and cost-effective, pediatric *Tuina* is widely used in China and is more easily accepted by children than other kinds of treatment, such as medication [9]. Based on TCM *Zang-Fu organ* theory and meridian theory, pediatric *Tuina* is used to treat common diseases in children by using a series of manual manipulation techniques at specified locations on the surface of the body [10]. Most acupoints of children are located primarily on the fingers, palms, arms, head, abdomen and back and include points such as *yiwofeng, neibagua,* and *liufu*, which are different from the standard human acupoints in adults [11].

Early studies have suggested potential benefits of pediatric *Tuina* in relieving symptoms of acute diarrhea and reducing the incidence of acute diarrhea [12–15]. Some researchers found that *Tuina*, massage or other similar touch therapy on children might adjust the level of cortical hormone, dopamine or insulin to accelerate the blood and lymph circulation, improve the function of the gastrointestinal system, or reduce the level of inflammatory mediators [16–20]. The evidence of an effect of pediatric *Tuina* compared with controls is inclusive. In a meta-analysis by Li Gao et al., pediatric massage showed a significantly better effect in terms of the clinical effective rate, clinical cure rate, and cure time than pharmacotherapy among children with acute diarrhea [21]. Another meta-analysis demonstrated that pediatric *Tuina* was superior to conventional medicine in improving the clinical cure rate and in decreasing the duration of acute diarrhea and daily stool frequency in children under 5 years old [22]. However, the low methodological quality, such as inappropriate random sequence generation and allocation concealment, as well as imperfect blinding in these studies, weakened the validity of their findings [23] and hampered widespread application of *Tuina* in clinical practice. Few well-designed randomized controlled trials (RCTs) have been performed to demonstrate the effect of pediatric *Tuina* among children with acute diarrhea. Therefore, the purpose of this trial was to assess the effect of pediatric *Tuina,* compared with sham *Tuina,* as an add-on therapy in addition to usual care for 0–6-year-old children with acute diarrhea. The hypothesis of our study was that pediatric *Tuina* can reduce times of diarrhea and/or shorten the duration of diarrhea.

## Methods

### Study design

The randomized, double-blind, sham-controlled trial was designed by Guangdong Provincial Hospital of Chinese Medicine and conducted in Dongguan Kanghua Hospital in Guangdong Province, China, from January 2017 to October 2019. The trial was approved by both ethics committees at Guangdong Provincial Hospital of Chinese Medicine and Dongguan Kanghua Hospital. The trial has been registered at ClinicalTrials.gov (NCT03005821), and the study protocol has been published [24]. Written informed consent was obtained from all participants’ guardians or parents by trained investigators. Oral informed consent was also obtained when the children were between 3 and 6 years of age.

### Participants

Participant recruitment was performed in Dongguan Kanghua Hospital in Guangdong Province, China, from January 2017 to October 2019. Inclusion criteria included 1) children aged 0–6 years; 2) the first occurrence of diarrhea within 72 h;3) children who met the acute diarrhea diagnostic criteria identified by *Shen* and *Wang* [25] and the WHO [26]; 4) stool frequency equal to or greater than 5 times per day; 5) no concurrent involvement in any other clinical trial; 6) guardians or parents able to coordinate care during the clinical trial; and 7) written informed consent signed by children’s guardians or parents. Exclusion criteria included 1) a history of Chinese pediatric *Tuina* on hands; 2) diagnosis of cholera or malaria; 3) children with any of the following conditions on the area to be manipulated: phlebitis, open wound, fracture, or tissue damage; and 4) children with any of the following complications: severe dehydration, metabolic acidosis, disorders of consciousness, seizures, twitching, shock, or azotemia. Demographic and clinical characteristics data were obtained through questionnaires in a separate room at each visit.

### Randomization and masking

Participants were randomly allocated in a 1:1 ratio to receive pediatric *Tuina* or sham *Tuina* by using a computer-generated random sequence. Allocation concealment was ensured by sealed envelopes generated by an independent statistician and was not available to any member of the research team until completion of analysis. The patients, caregivers, research staff, outcome assessors and data statisticians were all blinded to group assignment. *Tuina* therapists were not blinded, as sham *Tuina* was practiced differently from real pediatric *Tuina*. However, *Tuina* therapists were not allowed to share any information in terms of patients’ intervention with either patients, guardians/parents or any researchers, including outcome assessors and data statisticians.

To ensure successful blinding implementation, the therapists performed manipulations under an opaque cloak-shaped device [24], which was large enough to cover the upper body and arms of a child who was 6 years old or younger. Two narrow holes were made for therapists to insert their hands through and perform *Tuina*.

### Interventions

The participants in both groups received usual care, including montmorillonite powder and racecadotril granules. Intravenous infusion, ORS, zinc or antibiotics were administered if the pediatrician in charge considered it necessary.

In addition to usual care, eligible children received either Chinese pediatric *Tuina* or sham pediatric *Tuina* for 15–25 min, once per day, for 3 consecutive days. Follow-up evaluations were performed on day 7 and day 14.

**Fig. 1:**
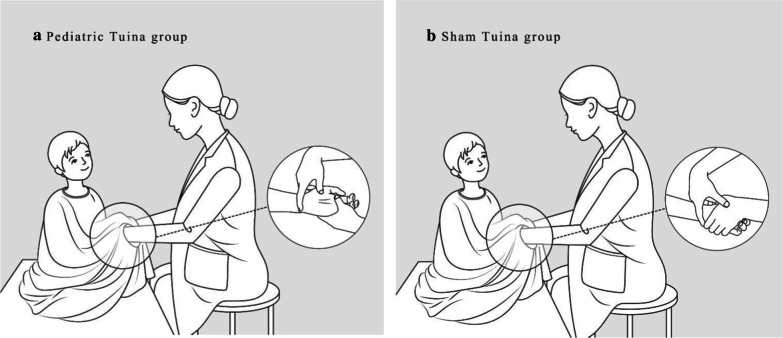
Pediatric *Tuina* manipulation methods for *Tuina* group (**a**) and for Sham *Tuina* group (**b**)

Two qualified therapists were involved in this trial, one of them had 2 years’ and the other had 3 years’ experiences in pediatric *Tuina* practice. Before the manipulation, all therapists were trained to ensure identical *Tuina* treatment according to standard operating procedures. When receiving therapy, the older children sat either on a bed or on a stool, and the children under 2 years old sat on their caregiver’s lap. *Tuina* treatment in the intervention group was performed on the surface of the children’s body using moderate pressure (Fig. 1a). *Tuina* treatment in the control group was different: the therapist used one hand to hold the child’s hand or put one hand on the child’s body, while the other hand performed manipulations on the therapist’s own hand instead of the child’s hand or body (Fig. 1b).

The standardized *Tuina* therapy prescription for two groups included six acupoints suggested by a panel of five pediatric *Tuina* experts who had at least 10 years of clinical experience in this area. Manipulation times for each acupoint depended on the age of the child. Detailed information on the *Tuina* acupoints, manipulation times and methods is provided in Additional file 1: Table 1.

### Outcomes

The primary outcomes were 1) days of diarrhea from baseline and 2) times of diarrhea on day 3. Days of diarrhea referred to the number of days from baseline to the first day that the frequency of diarrhea was reduced to equal to or less than twice per day within the treatment and follow-up period. Times of diarrhea were evaluated on day 3 of intervention. In this study, we defined the baseline and the 1st day of intervention as the same day.

Secondary outcomes included the following: 1) A global change rating (GCR): the GCR described a patient’s overall clinical state as a “global impression” as independently obtained by the assessor and was evaluated on day 7 and day 14 from baseline. The GCR was based on a 5-point scale as follows: “much better”, “slightly better”, “unchanged”, “slightly worse”, and “much worse”. It was applied as an assessor-assistant parents reported outcome, i.e. in the absence of research staff and *Tuina* therapists, the assessor read out the sentence of GCR word-by-word to the child’s parents and recorded according to their choices. As no one reported “slightly worse” or “much worse”, we further combined the categories into “much better” and “slightly better or unchanged”; 2) number of days when the stool characteristics returned to normal: the number of days from baseline to the first day that the stool characteristics returned to normal within the treatment and follow-up period. In addition, treatment-emergent adverse events (AEs) that occurred during the entire *Tuina* treatment were collected by AE report forms. The Principal Investigator and the Institutional Review Board who were blinded to treatment allocation reviewed all adverse events.

Two assessors were involved in this study. They were trained according to the *Tuina* protocols before the study started. The inter-observer reliability was good.

### Covariates

We collected information via questionnaires on demographic and clinical characteristics, including the child’s age, sex, height, weight, medical history, birth characteristics, allergy, feeding methods, clinical symptoms and types of treatment/medication before randomization and details of usual care during the study period in the current disease.

### Sample size

The sample size of this study was estimated based on the two primary outcome measures. Estimation according to diarrhea days was based on results from previous studies [27], which established diarrhea days in children as 2.1 days after receiving *Tuina* plus usual care, and the estimated standard deviation was 1.4. Diarrhea days in children after receiving usual care was 4.0, and the estimated standard deviation was 3.5. We needed 70 participants to achieve 90% power at α = 0.05 (two-sided). Assuming a dropout rate of 20%, we needed to enroll 84 participants in total (42 in each group).

Estimation according to diarrhea times was likewise based on reference from previous studies [28], which established diarrhea times in children as 4.1 times per day after receiving *Tuina* plus usual care, and the estimated standard deviation was 1.5. Diarrhea times of children after receiving usual care were 6.2 times per day, and the estimated standard deviation was 4.8. We needed 102 participants to achieve 90% power at α = 0.05 (two-sided). Assuming a dropout rate of 20%, we enrolled 122 participants in total (61 in each group).

Based on these results, we planned to choose the larger sample size (122 participants) for the estimated final trial sample size.

### Statistical analysis

Social demographic and clinical characteristics of the participants were presented as number (%) for categorical variables, mean (standard deviation, SD) for normally distributed continuous variables, and media (interquartile range, IQR) for nonnormally distributed continuous variables. Group comparisons were performed using *t* tests for continuous variables and chi-square tests or Fisher’s exact test for binary or categorical variables.

According to the prespecified statistical analysis plan [24], the association of *Tuina* with primary and secondary outcomes was analyzed by the intention-to-treat (ITT) and per-protocol (PP) approaches, respectively. For ITT analysis, we included all participants after randomization, regardless of whether they completed the treatment or adhered to the protocol. For PP analysis, we only included the participants who complied with the whole treatment. For the primary outcomes and number of days when the stool characteristics returned to normal (i.e., count data outcomes), we used a Poisson regression model and reported risk ratios (RRs) with 95% CIs for the studied associations. For GCR (i.e., binary outcome), we used a logistic regression model and reported odds ratios (ORs) with 95% CIs for the studied associations. In all models, we adjusted for covariates that were described in the previous section.

To assess if the age-dependent manipulation times influence the studied associations, we conducted a supplementary analysis for children younger than 2 years (*n* = 63). Analyses for children ages 2- < 4 years (*n* = 16) and 4–6 years (*n* = 5) were not done due to lack of power.

Statistical analysis was performed using SAS version 9.4 (SAS Institute Inc., Cary, NC, USA). A two-sided *P* < 0.05 indicated statistical significance.

## Results

**Fig. 2:**
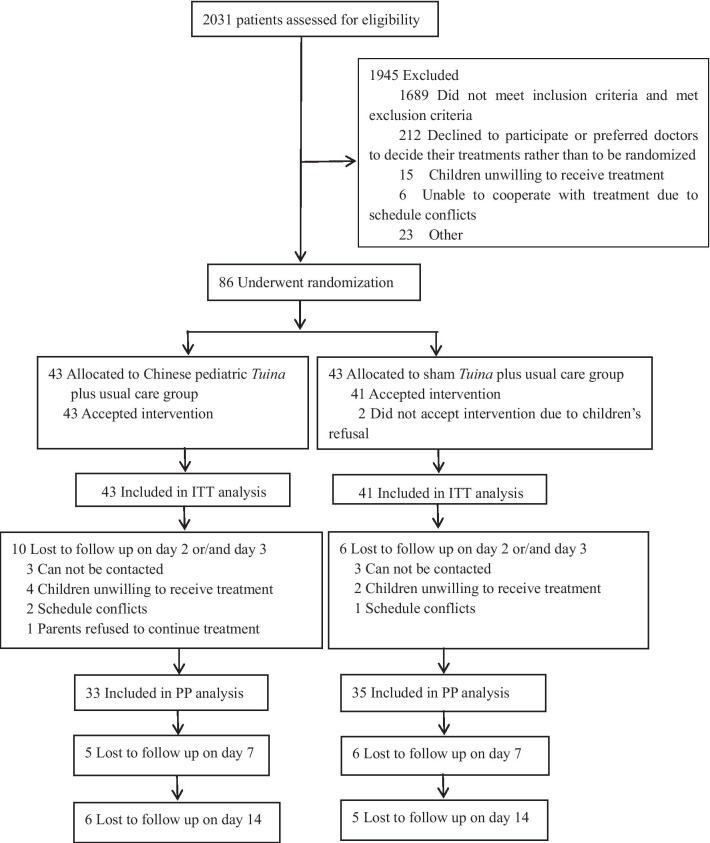
Consolidated Standards of Reporting Trials (CONSORT) Diagram for a Trial of pediatric *Tuina* on children with acute diarrhea

In total, 2031 children with acute diarrhea were screened at Dongguan Kanghua Hospital from January 8, 2017, through October 2019, of which 1689 were ineligible and 256 were eligible but could not be enrolled for a variety of reasons (Fig. 2). After exclusion, 86 children underwent randomization:43 children were randomized to the pediatric *Tuina* group and 43 to the sham *Tuina* group. Two children from the sham *Tuina* group did not accept *Tuina* intervention. A total of 84 children were included in the ITT analyses, whereas 68 patients were included in the PP analyses (33 in the pediatric *Tuina* group and 35 in the sham *Tuina* group) (Fig. 2). No difference was shown between the included and excluded patients in terms of the demographic and clinical characteristics (Additional file 1: Tables 2 and 3).

The baseline demographic and clinical characteristics were generally similar between the two groups (Table 1 and Additional file 1: Table 4). The mean (SD) age was 18.7 (14.3) months for all children. The hours of diarrhea before randomization, times of diarrhea during the past 24 h before randomization, and stool characteristics were balanced between the two groups.

**Table 1:** Baseline demographic characteristics of the participating children (*N* = 84)

Characteristics	All	Sham *Tuina* (***N*** = 41)	Pediatric *Tuina* (***N*** = 43)	***P*** value
Age (month, mean[SD])	18.7 (14.3)	19.0 (15.1)	18.4 (13.7)	0.87
0 - < 24 (N[%])	63 (75.00)	32 (78.05)	31 (72.09)	0.15
24 - < 48 (N[%])	16 (19.05)	5 (12.20)	11 (25.58)	
48–72 (N[%])	5 (5.95)	4 (9.76)	1 (2.33)	
Sex (N[%])				
Male	48 (57.14)	20 (48.78)	28 (65.12)	0.13
Female	36 (42.86)	21 (51.22)	15 (34.88)	
Height (cm)	79.1 (14.0)	78.3 (16.8)	79.8 (11.3)	0.66
Weight (kg)	10.6 (3.5)	10.5 (3.4)	10.8 (3.6)	0.68
History of neonatal disease (N[%])				
No	61 (72.62)	33 (80.49)	28 (65.12)	0.18
Yes	22 (26.19)	8 (19.51)	14 (32.56)	
Unknown/Missing	1 (1.19)	0 (0.00)	1 (2.33)	
History of infectious disease (N[%])				
No	73 (86.90)	35 (85.37)	38 (88.37)	0.68
Yes	11 (13.10)	6 (14.63)	5 (11.63)	
History of digestive disease (N[%])				
No	74 (88.10)	36 (87.80)	38 (88.37)	0.94
Yes	10 (11.90)	5 (12.20)	5 (11.63)	
History of respiratory disease (N[%])				
No	63 (75.00)	28 (68.29)	35 (81.40)	0.25
Yes	20 (23.81)	12 (29.27)	8 (18.60)	
Unknown/Missing	1 (1.19)	1 (2.44)	0 (0.00)	
History of other disease (N[%])				
No	68 (80.95)	31 (75.61)	37 (86.05)	0.22
Yes	16 (19.05)	10 (24.39)	6 (13.95)	
Mode of delivery (N[%])				
Vaginal	48 (57.14)	24 (58.54)	24 (55.81)	0.80
Cesarean	36 (42.86)	17 (41.46)	19 (44.19)	
Gestational age (N[%])				
Term	80 (95.24)	41 (100.00)	39 (90.70)	0.12
Preterm	4 (4.76)	0 (0.00)	4 (9.30)	
Birth weight (g)	3282.2 (451.9)	3274.3 (369.8)	3289.5 (521.1)	0.88
Order of delivery (N[%])				
First	40 (47.62)	20 (48.78)	20 (46.51)	1.00
Second	42 (50.00)	20 (48.78)	22 (51.16)	
Third	2 (2.38)	1 (2.44)	1 (2.33)	
Food allergy (N[%])				
No	76 (90.48)	35 (85.37)	41 (95.35)	0.06
Yes	7 (8.33)	6 (14.63)	1 (2.33)	
Missing	1 (1.19)	0 (0.00)	1 (2.33)	
Feeding (for children < 2 years of age, *N* *= 63) (N[%])*				
Breast	6 (9.52)	4 (12.50)	2 (6.45)	0.22
Mixed	20 (31.75)	7 (21.88)	13 (41.94)	
Milk	35 (55.56)	19 (59.38)	16 (51.61)	
Missing	2 (3.17)	2 (6.25)	0 (0.00)	
Complementary food (for children < 2 years of age, *N* *= 63) (N[%])*				
No	9 (14.29)	6 (18.75)	3 (9.68)	0.08
Yes	50 (79.37)	26 (81.25)	24 (77.42)	
Missing	4 (6.35)	0 (0.00)	4 (12.90)	

*P*-values based on T-test for continuous variable and Chi-square test or Fisher’s exact test (when expected cell counts less than 5) for binary or categorical variable

*SD* Standard deviation

Additionally, the usual care and concomitant treatments that were administered during the study period were balanced between the two groups, with one exception: the sham *Tuina* group received Chinese patent medicine more frequently than the pediatric *Tuina* group did on day 2 (Additional file 1: Tables 5 and 6).

Table 2 shows the results of the ITT analysis. Pediatric *Tuina* was associated with reduced times of diarrhea on day 3 (crude RR, 0.73 [95% CI, 0.59–0.91]) compared with sham *Tuina*. However, the association was alleviated to null after adjustment for social demographic and clinical characteristics (adjusted RR, 0.82 [95% CI, 0.64–1.04]). The days of diarrhea from baseline did not differ statistically between the pediatric *Tuina* group and sham *Tuina* group (adjusted RR, 1.02 [95% CI, 0.80–1.29]). For the GCR, we found no significant effect on day 7 (adjusted OR, 0.58 [95% CI, 0.17–1.92]) or on day 14 (adjusted OR, 1.06 [95% CI, 0.31–3.66]). The number of days when the stool characteristics returned to normal (adjusted RR, 0.95 [95% CI, 0.79–1.15]) was not significantly reduced. The analyses for children younger than 2 years showed similar results (Additional file 1: Table 7).

**Table 2:** Association between intervention and outcomes among the participating children (*N* = 84, Intention-to-treat analysis)

Outcome	All	Sham *Tuina* (***N*** = 41)	Pediatric *Tuina* (***N*** = 43)
Times of diarrhea on day 3			
Mean (SD)	4.7 (4.1)	5.5 (4.7)	4.0 (3.2)
Median (IQR)	4 (2,7)	4 (3,8)	4 (2,6)
Crude RR (95%CI)		Ref	**0.73 (0.59–0.91)**
Adjusted RR (95%CI)^a^		Ref	0.82 (0.64–1.04)
Days of diarrhea from baseline			
Mean (SD)	4.3 (3.2)	4.5 (3.1)	4.1 (3.3)
Median (IQR)	3 (2,6)	3.5 (2,6)	3 (2,6)
Crude RR (95%CI)		Ref	0.92 (0.74–1.16)
Adjusted RR (95%CI)^b^		Ref	1.02 (0.80–1.29)
Evaluation at day 7			
Much better (N[%])	53 (63.1)	28 (68.3)	25 (58.1)
Slightly better or no change (ref) (N[%])	20 (34.5)	7 (17.0)	13 (30.2)
Missing (N[%])	11 (13.1)	6 (14.6)	5 (11.2)
Crude OR(95%CI)		Ref	0.48 (0.17–1.40)
Adjusted OR (95%CI)^b^		Ref	0.58 (0.17–1.92)
Evaluation at day 14			
Much better (N[%])	56 (66.7)	27 (65.9)	29 (67.4)
Slightly better or no change (ref) (N[%])	17 (20.2)	9 (22.0)	8 (18.6)
Missing (N[%])	11 (13.1)	5 (12.2)	6 (14.0)
Crude OR(95%CI)		Ref	1.21 (0.41–3.58)
Adjusted OR (95%CI)^b^		Ref	1.06 (0.31–3.66)
Number of days when the stool characteristics returned to normal			
Mean (SD)	6.7 (3.6)	6.9 (3.8)	6.6 (3.5)
Median (IQR)	7 (4,7)	7 (4,7)	7 (3,7)
Crude RR(95%CI)		Ref	0.96 (0.81–1.15)
Adjusted RR (95%CI)^b^		Ref	0.95 (0.79–1.15)

Risk ratios were estimated from Poisson Regression

Odds ratios were estimated from Logistic Regression

*SD* Standard deviation, *IQR* Interquartile range

^a^Model adjusted for age, sex, history of infectious and digestive diseases, food allergy, hours of diarrhea before randomization, numbers of treatments/medications before randomization, numbers of the symptoms before randomization, times of diarrhea during the past 24 h before randomization, times of diarrhea on Day 2, numbers of usual care from Day 1 to Day 2, and concomitant treatment from Day 1 to Day 2

^b^Model adjusted for age, sex, history of infectious and digestive diseases, food allergy, hours of diarrhea before randomization, numbers of treatments/medications before randomization, numbers of the symptoms before randomization, numbers of usual care from Day 1 to Day 3, and concomitant treatment from Day 1 to Day 3

The results of the PP analysis are presented in Table 3. The risk of diarrhea was lower in the pediatric *Tuina* group than that in the sham *Tuina group* (crude RR, 0.66 [95% CI, 0.53–0.83]) on day 3. However, when adjusted for social demographic and clinical characteristics, the association was alleviated to null (adjusted RR, 0.84 [95% CI, 0.65–1.09]). The days of diarrhea from baseline were 4.1 days in the pediatric *Tuina* group and 4.7 days in the sham *Tuina* group (adjusted RR, 0.96 [95% CI, 0.75–1.25]). No difference was observed in the GCR between the two groups on day 7 (adjusted OR, 0.63 [95% CI, 0.18–2.25]) or on day 14 (adjusted OR, 1.38 [95% CI, 0.34–5.53]). No group difference was observed in the number of days when the stool characteristics returned to normal (adjusted RR, 0.95 [95% CI, 0.78–1.16]). Similar results were shown among children younger than 2 years (Additional file 1: Table 8).

**Table 3:** Association between intervention and outcomes among the participating children (*N* = 68, Per-protocol analysis)

Outcome	All	Sham *Tuina* (***N*** = 35)	Pediatric *Tuina* (***N*** = 33)
Times of diarrhea on day 3			
Mean (SD)	4.8 (4.2)	5.7 (4.7)	3.8 (3.2)
Median (IQR)	4 (2,6.5)	4 (3,8)	3 (2,6)
Crude RR (95%CI)		Ref	**0.66 (0.53–0.83)**
Adjusted RR (95%CI)^a^		Ref	0.84 (0.65–1.09)
Days of diarrhea from baseline			
Mean (SD)	4.4 (3.4)	4.7 (3.2)	4.1 (3.6)
Median (IQR)	3 (2,6)	4 (3,6)	3 (2,6)
Crude RR (95%CI)		Ref	0.87 (0.69–1.11)
Adjusted RR (95%CI)^b^		Ref	0.96 (0.75–1.25)
Evaluation at day 7			
Much better (N[%])	47 (69.1)	25 (71.4)	22 (66.7)
Slightly better or no change (ref) (N[%])	18 (26.5)	7 (20.0)	11 (33.3)
Missing (N[%])	3 (4.4)	3 (8.6)	0 (0.0)
Crude OR(95%CI)		Ref	0.56 (0.19–1.69)
Adjusted OR (95%CI)^b^		Ref	0.63 (0.18–2.25)
Evaluation at day 14			
Much better (N[%])	49 (72.1)	24 (68.6)	25 (75.8)
Slightly better or no change (ref) (N[%])	15 (22.1)	9 (25.7)	6 (18.2)
Missing (N[%])	4 (5.9)	2 (5.7)	2 (6.1)
Crude OR(95%CI)		Ref	1.56 (0.48–5.06)
Adjusted OR (95%CI)^b^		Ref	1.38 (0.34–5.53)
Number of days when the stool characteristics returned to normal			
Mean (SD)	7.0 (3.7)	7.1 (3.8)	6.9 (3.7)
Median (IQR)	7 (4,7)	7 (4,8)	7 (4,7)
Crude RR (95%CI)		Ref	0.97 (0.81–1.16)
Adjusted RR (95%CI)^b^		Ref	0.95 (0.78–1.16)

*SD* Standard deviation, *IQR* Interquartile range

Risk ratios were estimated from Poisson Regression

Odds ratios were estimated from Logistic Regression

^a^Model adjusted for age, sex, history of infectious and digestive diseases, food allergy, hours of diarrhea before randomization, numbers of treatments/medications before randomization, numbers of the symptoms before randomization, times of diarrhea during the past 24 h before randomization, times of diarrhea on Day 2, numbers of usual care from Day 1 to Day 2, and concomitant treatment from Day 1 to Day 2

^b^Model adjusted for age, sex, history of infectious and digestive diseases, food allergy, hours of diarrhea before randomization, numbers of treatments/medications before randomization, numbers of the symptoms before randomization, numbers of usual care from Day 1 to Day 3, and concomitant treatment from Day 1 to Day 3

Adverse events were reported for three children who were all from the sham *Tuina* group. These three children were recruited into our study when they visited the outpatient department, though they had fever or vomiting symptoms in addition to the diarrhea diagnosis. Since their symptoms were not relieved after 1 day of treatment, the pediatricians decided that they should be hospitalized. Adverse events reported for all three cases were due to hospitalizations. According to the medical expert committee’s judgment, none of these hospitalizations were related to the use of pediatric *Tuina*. The children were followed up. After 3 days of treatment for 2 patients and 8 days for 1 patient, they recovered and were discharged without complications.

## Discussion

Our study found that as an add-on therapy based on usual care, pediatric *Tuina*, using both ITT and PP analyses, was more effective than sham pediatric *Tuina* in terms of reducing times of diarrhea on day 3 of the study period. No difference was observed in days of diarrhea, global change rating, or number of days until the stool characteristics returned to normal between the two groups. To our knowledge, this is the first randomized trial adopting sham pediatric *Tuina* as a placebo control to achieve a double-blind study.

Pediatric *Tuina* involved lots of acupoints. According to the meridian theory, these acupoints were related to a specific organ. It could help to normalise impaired functions and balance *Yin* and *Yang* of *Zang-Fu* organ by stimulating these specific acupoints [29]. In children with acute diarrhea, the gastrointestinal system was weak. *Tuina* therapy could promote gastric juice secretion, regulate gastrointestinal motility, and enhance gastrointestinal digestion and absorption function [30, 31]. Some studies demonstrated that pediatric *Tuina* used alone or combined with conventional treatment was effective for acute diarrhea compared with no treatment or other conventional treatment [32–35]. However, most of these studies had difficulties in interpretation due to methodological limitations. A meta-analysis demonstrated that pediatric *Tuina* was superior to conventional medication in improving the clinical cure rate and decreasing the duration of disease and daily stool frequency in children under 5 years old [22]. Other systematic reviews also showed that pediatric *Tuina* was more effective than conventional treatments [36, 37]. However, these studies only reported the overall effective rate but not detailed components of the outcome assessment, such as diarrhea days and diarrhea times.

As pediatric *Tuina* comes in the form of manipulation, it has been a challenge to develop and validate placebo control. No well-recognized sham control has been developed to date. The majority of studies [38–41] on *Tuina* evaluation used another kind of intervention when their effects were not confirmed as a control, which might cause challenges in the interpretation of *Tuina’s* effects [42]. Although some researchers used sham massage (i.e., gently still touch, producing no indentation on the skin) as a sham control [43, 44], the gentle touch procedure applied for sham massage [45] is similar to rubbing manipulation of traditional medicine, which might have a minor effect that may reduce the effect of *Tuina*, especially in the area of pediatric *Tuina*. As all the acupoints in our study were located on the upper body of the child, we designed an opaque cloak-shaped device to cover this part of the body. This cloak-shaped device, in addition to the sham manipulation, makes blinding for children, parents/guardians, and outcome assessors as well as the placebo-controlled design possible. It can exclude the possibility of an effect of light/gentle rubbing touch that the previous methods may have caused.

This study has several strengths. First, we recruited patients either from the outpatient or inpatient department, which might enlarge our study’s participant resources and ensure sufficient representation of the study population. Second, we designed an opaque cloak-shaped device to cover the upper body and arms of the child, which made the blinding design possible and minimized performance biases. Third, we used the sham *Tuina* as a control. On the one hand, use of a sham is very helpful for the interpretation of *Tuina’s* effects and can highlight the specificity of acupoints. On the other hand, it can improve the quality of RCTs and promote the international recognition of pediatric *Tuina*. The last strength is that the trial was rigorously conducted according to a prespecified protocol without changes, and the blinding was intact in both groups.

This study has some limitations. We screened 2031 children with acute diarrhea but failed to obtain the 122 eligible participants that we targeted, although a sample size of 84 met what we estimated for one primary outcome [24]. This may have compromised the statistical power in our analysis. A possible reason for insufficient recruitment might be that we adopted rather rigorous inclusion criteria, such as the stool frequency being equal to or more than 5 times a day, which led to the exclusion of 636 children, and 319 children could not be enrolled because the occurrence of diarrhea was not within 72 h. The usual care administered in the outpatient department might vary from that in the inpatient department, which might increase the heterogeneity and reduce the comparability between the two groups. Such confounding effects have, however, been partly controlled for by including the number of usual care treatments as a covariate in the analysis. In addition, this study was only performed in one center and the generalisability was limited. In the future, multicenter randomized controlled trials with larger sample size may further explore the efficacy of pediatric *Tuina* on children with acute diarrhea.

## Conclusions

In children aged 0–6 years with acute diarrhea, pediatric *Tuina* showed significant effects in terms of reducing times of diarrhea compared with sham *Tuina*. Studies with larger sample sizes and adjusted trial designs are warranted to further evaluate the effect of pediatric *Tuina* therapy.

## Supplementary Information

**Additional file 1.** **Table 1**. *Tuina* acupoints, manipulation times and methods. **Table 2.** Comparison of baseline demographic characteristics between the included and excluded children. **Table 3.** Comparison of baseline clinical characteristics between the included and excluded children. **Table 4.** Baseline clinical characteristics of the participating children (*N* = 84). **Table 5.** Usual care from Day 1 to Day 3 among the participating children (*N* = 84). **Table 6.** Concomitant treatment from Day 1 to Day 3 among the participating children (*N* = 84). **Table 7.** Association between intervention and outcomes among the children younger than 2 years (*N* = 63, Intention-to-treat analysis). **Table 8.** Association between intervention and outcomes among the children younger than 2 years (*N* = 49, Per-protocol analysis).

## Data Availability

All the data obtained and materials analyzed in this study are available with the corresponding author upon request.

## Abbreviations

AEs: Adverse events
GCR: Global change rating
IQR: Interquartile range
ITT: Intention-to-treat
ORS: Oral rehydration solution
ORs: Odds ratios
PP: Per-protocol
RCTs: Randomized control trials
RRs: Risk ratios
SD: Standard deviation
TCM: Traditional Chinese medicine
WHO: World Health Organization

## References

[1] Das JK, Bhutta ZA (2016). Global challenges in acute diarrhea. Curr Opin Gastroenterol.

[2] Walker CL, Rudan I, Liu L (2013). Global burden of childhood pneumonia and diarrhoea. Lancet.

[3] GBD 2015 Mortality and Causes of Death Collaborators (2016). Global, regional, and national life expectancy, all-cause and cause-specific mortality for 249 causes of death, 1980–2015: a systematic analysis for the global burden of disease study 2015. Lancet.

[4] GBD Diarrhoeal Diseases Collaborators (2017). Estimates of global, regional, and national morbidity, mortality, and aetiologies of diarrhoeal diseases: a systematic analysis for the global burden of disease study 2015. Lancet Infect Dis.

[5] Simpson E, Zwisler G, Moodley M (2013). Survey of caregivers in Kenya to assess perceptions of zinc as a treatment for diarrhea in young children and adherence to recommended treatment behaviors. J Glob Health.

[6] Ahmed S, Nasrin D, Ferdous F (2013). Acceptability and compliance to a 10-day regimen of zinc treatment in diarrhea in rural Bangladesh. Food Nutr Sci.

[7] Donowitz M, Alpers DH, Binder HJ, Brewer T, Carrington J, Grey MJ (2012). Translational approaches for pharmacotherapy development for acute diarrhea. Gastroenterology.

[8] Zwisler G, Simpson E, Moodley M (2013). Treatment of diarrhea in young children: results from surveys on the perception and use of oral rehydration solutions, antibiotics, and other therapies in India and Kenya. J Glob Health.

[9] Zhang XH, Guo TP, Zhu BW (2018). Pediatric Tuina for promoting growth and development of preterm infants: a protocol for the systematic review of randomized controlled trail. Medicine (Baltimore).

[10] World Health Organization (2010). Benchmarks for training in traditional/ complementary and alternative medicine:benchmarks for training in tuina.

[11] Liao PD (2016). Pediatric Tuina [in Chinese].

[12] Liu JE (2018). Comparative analysis on clinical curative effects of acupuncture combined with Chinese massage and montmorillonite powder in treatment of children with acute non-bacterial diarrhea [in Chinese]. J Basic Chin Med.

[13] Liu L, Yang WT, Zheng YL (2015). Comparative analysis on clinical curative effects of traditional Chinese medicine decoction combined with *Tuina* and montmorillonite powder in treatment of children with acute non-bacterial diarrhea [in Chinese]. Mater Child Health Care China.

[14] Wang KT, Li QR (2017). Therapeutic effect of *Tuina* combined with *Nuanqitie*on children with acute non-bacterial diarrhea [in Chinese]. Mater Child Health Care China.

[15] Chang ZH, Chen CL, Cheng K (2019). Study on the therapeutic effect of three-character-scripture school *Tuina* combined with acupuncture in the treatment of acute diarrhea in children [in Chinese]. Lab Med Clin.

[16] Field T, Hernandez-Reif M, Diego M, Schanberg S, Kuhn C (2005). Cortisol decreases and serotonin and dopamine increase following massage therapy. Int J Neurosci.

[17] Field T, Diego M, Hemandez-Reif M (2008). Insulin and insulin-like growth factor-1 increased in preterm neonates following massage therapy. J Dev Behav Pediatr.

[18] Lindgren L, Rundgren S, Winsö O (2010). Physiological responses to touch massage in healthy volunteers. Auton Neurosci.

[19] Powell L, Cheshire A, Swaby L (2010). Children’s experiences of their participation in a training and support programme involving massage. Complement Ther Clin Pract.

[20] Zhou HY, Xu XM (2020). Effect of TCM Tuina on immune function and inflammatory factors in children with diarrhea [in Chinese]. J Pract Tradit Chin Intern Med.

[21] Gao L, Jia C, Huang H (2018). Paediatric massage for treatment of acute diarrhoea in children: a meta-analysis. BMC Complement Altern Med.

[22] Lai BY, Liang N, Cao HJ (2018). Pediatric Tui Na for acute diarrhea in children under 5 years old: a systematic review and meta-analysis of randomized clinical trials. Complement Ther Med.

[23] Lai BY, Wang LQ, Jia LY (2018). Evaluation on methodological and reporting quality of randomized controlled trials of Tuina therapy for childhood diarrhea using CONSORT and STRICTA standards [in Chinese]. J Tradit Chin Med.

[24] Lu TY, Zhang HY, Yin LJ (2019). Chinese pediatric *Tuina* on children with acute diarrhea: study protocol for a randomized sham-controlled trial. Trials.

[25] Shen XM, Wang WP (2011). Pediatrics [in Chinese].

[26] World Health Organization (2015). Diarrhoeal disease fact sheet N 330.

[27] Huang YL, Lv FW (1999). Treating 35 cases of infantile autumn diarrhea by Smecta and Tuina [in Chinese]. Shandong J Tradit Chin Med.

[28] Li Y, Di W (2006). Observation of therapeutic effect of massage on rotavirus enteritis [in Chinese]. Modern J Integr Tradit Chin Western Med.

[29] Chen LL, Su YC, Su CH, Lin HC, Kuo HW (2008). Acupressure and meridian massage: combined effects on increasing body weight in premature infants. J Clin Nurs.

[30] Yang XX (2005). Observation on therapeutic effect of Tuina on infantile diarrhea [in Chinese]. Liaoning J Tradit Chin Med.

[31] Tian QL (2008). 55 cases of infantile diarrhea treated with the combination of Tuina and auricular point sticking [in Chinese]. Shanghai J Acup Moxib.

[32] Xie WJ, Li WC, Tang W, Liu SY (2019). Clinical observation of Liu’s pediatric Tuina in the treatment of acute diarrhea in children [in Chinese]. J Pract Tradit Chin Med..

[33] Cui XL (2019). Observation on therapeutic effect of moxibustion combined with *Tuina* on children with acute diarrhea [in Chinese]. J Pract Tradit Chin Med.

[34] Shang Y (2018). Clinical observation on 45 cases of acute non-bacterial infections diarrhea in children treated with the combination of modified Weiling decoction and Tuina [in Chinese]. J Pediatr Tradit Chin Med.

[35] Li XF, Yan YB, Dian YB (2015). Clinical effective observation on treating diarrhea in the acute phase in infants by massage [in Chinese]. Clin J Chin Med.

[36] Guo XQ, Wang YG, Zeng QY (2012). Systematic review of pediatric tuina for infantile diarrhea [in Chinese]. J Tradit Chin Med.

[37] Si MR, Cai JH, Wang WY, Ji Q (2016). Systematic review of comparing pediatric tuina therapy with montmorillonite in treating children diarrhea [in Chinese]. China Med Herald.

[38] Peng Y, Leng L, Chen Z (2011). Acute infantile diarrhea treated with infantile Tuina: a multicentre randomized controlled trial [in Chinese]. Chin Acup Moxib.

[39] Tao HQ, Li ZS, Xu L (2015). Clinical efficacy of massage in treatment of infantile indigestion diarrhea caused by improper diet: a report of 30 cases [in Chinese]. J Anhui Univ Chin Med.

[40] Chen QY, Li RH, Wang AJ (2019). Clinical study of traditional Chinese medicine massage therapy in the treatment of diarrhea in children [in Chinese]. Res J Integr Tradit Chin West Med.

[41] Tang ST, Zhang LL (2020). Massage therapy for 45 cases of chronic diarrhea in children [in Chinese]. Chin J Trait Med Sci Techno.

[42] Wang YJ, Guo Y (2009). Study on key problems in the positive control design methods of acupuncture and moxibustion [in Chinese]. Chin Acup Moxib.

[43] Ho YB, Lee RS, Chow CB, Pang MY (2010). Impact of massage therapy on motor outcomes in very low-birthweight infants: randomized controlled pilot study. Pediatr Int.

[44] Diego MA, Field T, Hernandez-Reif M (2005). Vagal activity, gastric motility, and weight gain in massaged preterm neonates. J Pediatr.

[45] Vickers A, Ohlsson A, Lacy JB, Horsley A. Massage for promotinggrowth and development of preterm and/or low birth-weight infants. https://www.cochrane.org/CD000390. Accessed 12 July 2020.

