# Supplementary material for: Chinese pediatric Tuina on children with acute diarrhea: a randomized sham-controlled trial
Source: Health Qual Life Outcomes. 2021 Jan 6;19:4. doi: 10.1186/s12955-020-01636-1 (PMC7788799; doi:10.1186/s12955-020-01636-1)
Supplement: Supplementary file 1 — Additional file 1. Table 1. Tuina acupoints, manipulation times and methods. Table 2. Comparison of baseline demographic characteristics between the included and excluded children. Table 3. Comparison of baseline clinical characteristics between the included and excluded children. Table 4. Baseline clinical characteristics of the participating children (N = 84). Table 5. Usual care from Day 1 to Day 3 among the participating children (N = 84). Table 6. Concomitant treatment from Day 1 to Day 3 among the participating children (N = 84). Table 7. Association between intervention and outcomes among the children younger than 2 years (N = 63, Intention-to-treat analysis). Table 8. Association between intervention and outcomes among the children younger than 2 years (N = 49, Per-protocol analysis). [file 12955_2020_1636_MOESM1_ESM.docx]

**Supplementary Table 1. *Tuina* acupoints, manipulation times and methods**

| Age | Total Times | | | | | |
| --- | --- | --- | --- | --- | --- | --- |
|  | *Neibagua* point-  arc-push,  clockwise^1^ | Small intestine meridian point-  clearing^2^ | Large intestine meridian point-  nourishing^3^ | *Banmen*  point-  kneading^4^ | Abdomen-  rubbing, anti-clockwise^5^ | *Qijiegu*  point-  pushing up^6^ |
| ＜2 years old (0-1 years old) | 500 | 300 | 300 | 300 | 300 | 300 |
| ≥2 years old and＜4 years old (2-3 years old) | 700 | 500 | 500 | 500 | 500 | 500 |
| ≥4 years old and ≤6 years old (4-6 years old) | 1000 | 800 | 800 | 800 | 800 | 800 |

Notes:

1. It is a circle point with the center of palm as the center and 2/3 of the center to the transverse crease of the middle finger root as the radius. In manipulation, arc-push this point clockwise*.

2. It is a linear point in the lateral side of the little finger, from its tip to root. In manipulation, push the lateral side of the little finger straight from the root to its tip for clearing*.

3. It is a linear point in the lateral side of the index finger, from its tip to root. In manipulation, push this point straight from the finger tip to the root for nourishing*.

4. It is a dotty point in the middle of the big thenar. In manipulation, press this point and knead it*.

5. It is a surface point on the belly. In manipulation, rubbing it anti-clockwise.

6. It is a linear point on the back along the spine from the fourth lumbar vertebra to the spinal end of tailbone. In manipulation, push along the spine from the spinal end of tailbone to the fourth lumbar vertebra for nourishing.

*Ge MF, Ye X. Chinese-English Edition of Three-Character-Scripture School Pediatric Massage. Shanghai China: Shanghai Scientific and Technical Publishers; 2008, p25, p27, p31.

| **Supplementary Table 2. Comparison of baseline demographic characteristics between the included and excluded children** | | | | |
| --- | --- | --- | --- | --- |
| **Characteristics** | **All** | **Included (N=68)** | **Excluded (N=16)** | **P value** |
| **Age (mean[SD])** | 18.7 (14.3) | 19.1 (14.7) | 17.0 (12.5) | 0.60 |
| **Sex (N[%])** |  |  |  |  |
| Male | 48 (57.14) | 39 (57.35) | 9 (56.25) | 0.94 |
| Female | 36 (42.86) | 29 (42.65) | 7 (43.75) |  |
| **Height (cm)** | 79.1 (14.0) | 79.0 (14.9) | 79.3 (10.6) | 0.94 |
| **Weight (kg)** | 10.6 (3.5) | 10.7 (3.7) | 10.5 (2.4) | 0.87 |
| **History of neonatal disease (N[%])** |  |  |  |  |
| No | 61 (72.62) | 49 (72.06) | 12 (75.00) | 0.19 |
| Yes | 22 (26.19) | 19 (27.94) | 3 (18.75) |  |
| Unknown/Missing | 1 (1.19) | 0 (0.00) | 1 (6.25) |  |
| **History of infectious disease (N[%])** |  |  |  |  |
| No | 73 (86.90) | 59 (86.76) | 14 (87.50) | 1.00 |
| Yes | 11 (13.10) | 9 (13.24) | 2 (12.50) |  |
| **History of digestive disease (N[%])** |  |  |  |  |
| No | 74 (88.10) | 59 (86.76) | 15 (93.75) | 0.68 |
| Yes | 10 (11.90) | 9 (13.24) | 1 (6.25) |  |
| **History of respiratory disease (N[%])** |  |  |  |  |
| No | 63 (75.00) | 51 (75.00) | 12 (75.00) | 1.00 |
| Yes | 20 (23.81) | 16 (23.53) | 4 (25.00) |  |
| Unknown/Missing | 1 (1.19) | 1 (1.47) | 0 (0.00) |  |
| **History of other disease (N[%])** |  |  |  |  |
| No | 68 (80.95) | 54 (79.41) | 14 (87.50) | 0.73 |
| Yes | 16 (19.05) | 14 (20.59) | 2 (12.50) |  |
| **Mode of delivery (N[%])** |  |  |  |  |
| Vaginal | 48 (57.14) | 40 (58.82) | 8 (50.00) | 0.52 |
| Cesarean | 36 (42.86) | 28 (41.18) | 8 (50.00) |  |
| **Gestational age (N[%])** |  |  |  |  |
| Term | 80 (95.24) | 65 (95.59) | 15 (93.75) | 0.58 |
| Preterm | 4 (4.76) | 3 (4.41) | 1 (6.25) |  |
| **Birth weight (g)** | 3282.2 (451.9) | 3244.3(410.5) | 3440.6 (584.3) | 0.12 |
| **Order of delivery (N[%])** |  |  |  |  |
| First | 40 (47.62) | 32 (47.06) | 8 (50.00) | 1.00 |
| Second | 42 (50.00) | 34 (50.00) | 8 (50.00) |  |
| Third | 2 (2.38) | 2 (2.94) | 0 (0.00) |  |
| **Food allergy (N[%])** |  |  |  |  |
| No | 76 (90.48) | 63 (92.65) | 13 (81.25) | 0.13 |
| Yes | 7 (8.33) | 5 (7.35) | 2 (12.50) |  |
| Unknown/Missing | 1 (1.19) | 0 (0.00) | 1 (6.25) |  |
| **Feeding (for children<2 years of age, N=63) (N[%])** |  |  |  |  |
| Breast | 6 (9.52) | 5 (10.20) | 1 (7.14) | 0.45 |
| Mixed | 20 (31.75) | 13 (26.53) | 7 (50.00) |  |
| Milk | 35 (55.56) | 29 (59.18) | 6 (42.86) |  |
| Missing | 2 (3.17) | 2 (4.08) | 0 (0.00) |  |
| **Complementary food (for children<2 years of age, N=63) (N[%])** |  |  |  |  |
| No | 9 (14.29) | 9 (18.37) | 0 (0.00) | 0.08 |
| Yes | 50 (79.37) | 38 (77.55) | 12 (85.71) |  |
| Missing | 4 (6.35) | 2 (4.08) | 2 (14.29) |  |
| SD: standard deviation |  |  |  |  |
| P-values based on T-test for continuous variable and Chi-square test or Fisher’s exact test (when expected cell counts less than 5) for binary or categorical variable | | | | |

| **Supplementary Table 3. Comparison of baseline clinical characteristics between the included and excluded children** | | | | |
| --- | --- | --- | --- | --- |
| **Characteristics** | **All** | **Included (N=68)** | **Excluded (N=16)** | **P value** |
| **Hours of diarrhea before randomization (mean[SD])** | 33.8 (15.6) | 34.2 (15.5) | 32.4 (16.3) | 0.68 |
| **Times of diarrhea during the past 24 hours before randomization (mean[SD])** | 8.8 (4.3) | 9.0 (4.6) | 7.8 (2.5) | 0.32 |
| **Stool characteristics (N[%])** |  |  |  |  |
| Shuiyang | 29 (34.52) | 23 (33.82) | 6 (37.50) | 0.94 |
| Danhua | 19 (22.62) | 16 (23.53) | 3 (18.75) |  |
| Xilan | 8 (9.52) | 6 (8.82) | 2 (12.50) |  |
| Nianye | 1 (1.19) | 1 (1.47) | 0 (0.00) |  |
| More than one characteristic | 27 (32.14) | 22 (32.35) | 5 (31.25) |  |
| **Stool color (N[%])** |  |  |  |  |
| Light yellow | 65 (77.38) | 54 (79.41) | 11 (68.75) | 0.53 |
| Green | 10 (11.90) | 8 (11.76) | 2 (12.50) |  |
| White | 1 (1.19) | 1 (1.47) | 0 (0.00) |  |
| More than one color | 8 (9.52) | 5 (7.35) | 3 (18.75) |  |
| **Stool smell (N[%])** |  |  |  |  |
| Less smelly than before | 21 (25.00) | 17 (25.00) | 4 (25.00) | 0.79 |
| Same as before | 17 (20.24) | 15 (22.06) | 2 (12.50) |  |
| More smelly than before | 46 (54.76) | 36 (52.94) | 10 (62.50) |  |
| **Fart severity (N[%])** |  |  |  |  |
| Mild | 33 (39.29) | 26 (38.24) | 7 (43.75) | 0.41 |
| Moderate or Severe | 14 (16.67) | 13 (19.12) | 1 (6.25) |  |
| Other | 35 (41.67) | 28 (41.18) | 7 (43.75) |  |
| Missing | 2 (2.38) | 1 (1.47) | 1 (6.25) |  |
| **Appetite (N[%])** |  |  |  |  |
| Good or average | 25 (29.76) | 20 (29.41) | 5 (31.25) | 1.00 |
| Poor | 59 (70.24) | 48 (70.59) | 11 (68.75) |  |
| **Dehydration (N[%])** |  |  |  |  |
| No | 60 (71.43) | 48 (70.59) | 12 (75.00) | 1.00 |
| Yes (Mild) | 22 (26.19) | 18 (26.47) | 4 (25.00) |  |
| Missing | 2 (2.38) | 2 (2.94) | 0 (0.00) |  |
| **Accompanying symptoms (N[%])** |  |  |  |  |
| ***Fever*** |  |  |  |  |
| No | 52 (61.90) | 42 (61.76) | 10 (62.50) | 1.00 |
| Yes | 31 (36.90) | 25 (36.76) | 6 (37.50) |  |
| Missing | 1 (1.19) | 1 (1.47) | 0 (0.00) |  |
| ***Vomiting*** |  |  |  |  |
| No | 49 (58.33) | 40 (58.82) | 9 (56.25) | 1.00 |
| Yes | 34 (40.48) | 27 (39.71) | 7 (43.75) |  |
| Missing | 1 (1.19) | 1 (1.47) | 0 (0.00) |  |
| ***Abdominal bloating*** |  |  |  |  |
| No | 65 (77.38) | 51 (75.00) | 14 (87.50) | 0.70 |
| Yes | 7 (8.33) | 6 (8.82) | 1 (6.25) |  |
| Missing | 12 (14.29) | 11 (16.18) | 1 (6.25) |  |
| ***Abdominal pain*** |  |  |  |  |
| No | 57 (67.86) | 47 (69.12) | 10 (62.50) | 0.47 |
| Yes | 17 (20.24) | 12 (17.65) | 5 (31.25) |  |
| Missing | 10 (11.90) | 9 (13.24) | 1 (6.25) |  |
| ***Cough*** |  |  |  |  |
| No | 75 (89.29) | 59 (86.76) | 16 (100.00) | 0.20 |
| Yes | 9 (10.71) | 9 (13.24) | 0 (0.00) |  |
| ***Runny nose*** |  |  |  |  |
| No | 79 (94.05) | 63 (92.65) | 16 (100.00) | 0.58 |
| Yes | 5 (5.95) | 5 (7.35) | 0 (0.00) |  |
| ***Numbers of the above symptoms (mean[SD])*** | 1.2 (1.1) | 1.2 (1.1) | 1.2 (1.0) | 0.88 |
| **Treatment/medications before randomization (N[%])** |  |  |  |  |
| ***Oral rehydration*** |  |  |  |  |
| No | 76 (90.48) | 62 (91.18) | 14 (87.50) | 0.64 |
| Yes | 8 (9.52) | 6 (8.82) | 2 (12.50) |  |
| ***Mucosal Protective Agents*** |  |  |  |  |
| No | 52 (61.90) | 44 (64.71) | 8 (50.00) | 0.28 |
| Yes | 32 (38.10) | 24 (35.29) | 8 (50.00) |  |
| ***Probiotics*** |  |  |  |  |
| No | 56 (66.67) | 44 (64.71) | 12 (75.00) | 0.56 |
| Yes | 28 (33.33) | 24 (35.29) | 4 (25.00) |  |
| ***Intravenous rehydration*** |  |  |  |  |
| No | 78 (92.86) | 64 (94.12) | 14 (87.50) | 0.32 |
| Yes | 6 (7.14) | 4 (5.88) | 2 (12.50) |  |
| ***Antibiotics or antivirus*** |  |  |  |  |
| No | 76 (90.48) | 60 (88.24) | 16 (100.00) | 0.34 |
| Yes | 8 (9.52) | 8 (11.76) | 0 (0.00) |  |
| ***Chinese patent medicine*** |  |  |  |  |
| No | 63 (75.00) | 49 (72.06) | 14 (87.50) | 0.34 |
| Yes | 21 (25.00) | 19 (27.94) | 2 (12.50) |  |
| ***Fever and pain reliever*** |  |  |  |  |
| No | 74 (88.10) | 61 (89.71) | 13 (81.25) | 0.39 |
| Yes | 10 (11.90) | 7 (10.29) | 3 (18.75) |  |
| ***Others*** |  |  |  |  |
| No | 48 (57.14) | 36 (52.94) | 12 (75.00) | 0.16 |
| Yes | 36 (42.86) | 32 (47.06) | 4 (25.00) |  |
| ***Numbers of the above Treatment/medications (mean[SD])*** | 1.8 (1.2) | 1.8 (1.2) | 1.6 (1.2) | 0.42 |
| SD: standard deviation |  |  |  |  |
| P-values based on T-test for continuous variable and Chi-square test or Fisher’s exact test (when expected cell counts less than 5) for binary or categorical variable | | | | |

| **Supplementary Table 4. Baseline clinical characteristics of the participating children (N=84)** | | | | |
| --- | --- | --- | --- | --- |
| **Characteristics** | **All** | **Sham Tuina(N=41)** | **Pediatric Tuina(N=43)** | **P value** |
| **Hours of diarrhea before randomization (mean[SD])** | 33.8 (15.6) | 35.0 (16.9) | 32.3 (14.4) | 0.51 |
| **Times of diarrhea during the past 24 hours before randomization (mean[SD])** | 8.8 (4.3) | 8.5 (4.6) | 9.0 (4.1) | 0.59 |
| **Stool characteristics (N[%])** |  |  |  |  |
| Shuiyang | 29 (34.52) | 10 (24.39) | 19 (44.19) | 0.21 |
| Danhua | 19 (22.62) | 11 (26.83) | 8 (18.60) |  |
| Xilan | 8 (9.52) | 3 (7.32) | 5 (11.63) |  |
| Nianye | 1 (1.19) | 1 (2.44) | 0 (0.00) |  |
| More than one characteristic | 27 (32.14) | 16 (39.02) | 11 (25.58) |  |
| **Stool color (N[%])** |  |  |  |  |
| Light yellow | 65 (77.38) | 31 (75.61) | 34 (79.07) | 0.61 |
| Green | 10 (11.90) | 6 (14.63) | 4 (9.30) |  |
| White | 1 (1.19) | 1 (2.44) | 0 (0.00) |  |
| More than one color | 8 (9.52) | 3 (7.32) | 5 (11.63) |  |
| **Stool smell (N[%])** |  |  |  |  |
| Less smelly than before | 21 (25.00) | 9 (21.95) | 12 (27.91) | 0.61 |
| Same as before | 17 (20.24) | 10 (24.39) | 7 (16.28) |  |
| More smelly than before | 46 (54.76) | 22 (53.66) | 24 (55.81) |  |
| **Fart severity (N[%])** |  |  |  |  |
| Mild | 33 (39.29) | 15 (36.59) | 18 (41.86) | 0.87 |
| Moderate or Severe | 14 (16.67) | 6 (14.63) | 8 (18.60) |  |
| Other | 35 (41.67) | 19 (46.34) | 16 (37.21) |  |
| Missing | 2 (2.38) | 1 (2.44) | 1 (2.33) |  |
| **Appetite (N[%])** |  |  |  |  |
| Good or average | 25 (29.76) | 10 (24.39) | 15 (34.88) | 0.29 |
| Poor | 59 (70.24) | 31 (75.61) | 28 (65.12) |  |
| **Dehydration (N[%])** |  |  |  |  |
| No | 60 (71.43) | 25 (60.98) | 35 (81.40) | 0.054 |
| Yes (Mild) | 22 (26.19) | 14 (34.15) | 8 (18.60) |  |
| Missing | 2 (2.38) | 2 (4.88) | 0 (0.00) |  |
| **Accompanying symptoms (N[%])** |  |  |  |  |
| ***Fever*** |  |  |  |  |
| No | 52 (61.90) | 24 (58.54) | 28 (65.12) | 0.50 |
| Yes | 31 (36.90) | 17 (41.46) | 14 (32.56) |  |
| Missing | 1 (1.19) | 0 (0.00) | 1 (2.33) |  |
| ***Vomiting*** |  |  |  |  |
| No | 49 (58.33) | 19 (46.34) | 30 (69.77) | **0.046** |
| Yes | 34 (40.48) | 21 (51.22) | 13 (30.23) |  |
| Missing | 1 (1.19) | 1 (2.44) | 0 (0.00) |  |
| ***Abdominal bloating*** |  |  |  |  |
| No | 65 (77.38) | 31 (75.61) | 34 (79.07) | 0.27 |
| Yes | 7 (8.33) | 2 (4.88) | 5 (11.63) |  |
| Missing | 12 (14.29) | 8 (19.51) | 4 (9.30) |  |
| ***Abdominal pain*** |  |  |  |  |
| No | 57 (67.86) | 27 (65.85) | 30 (69.77) | 0.94 |
| Yes | 17 (20.24) | 9 (21.95) | 8 (18.60) |  |
| Missing | 10 (11.90) | 5 (12.20) | 5 (11.63) |  |
| ***Cough*** |  |  |  |  |
| No | 75 (89.29) | 35 (85.37) | 40 (93.02) | 0.31 |
| Yes | 9 (10.71) | 6 (14.63) | 3 (6.98) |  |
| ***Runny nose*** |  |  |  |  |
| No | 79 (94.05) | 38 (92.68) | 41 (95.35) | 0.67 |
| Yes | 5 (5.95) | 3 (7.32) | 2 (4.65) |  |
| ***Numbers of the above symptoms (mean[SD])*** | 1.2 (1.1) | 1.4 (1.2) | 1.0 (1.0) | 0.12 |
| **Treatment/medications before randomization (N[%])** |  |  |  |  |
| ***Oral rehydration*** |  |  |  |  |
| No | 76 (90.48) | 37 (90.24) | 39 (90.70) | 1.00 |
| Yes | 8 (9.52) | 4 (9.76) | 4 (9.30) |  |
| ***Mucosal Protective Agents*** |  |  |  |  |
| No | 52 (61.90) | 26 (63.41) | 26 (60.47) | 0.78 |
| Yes | 32 (38.10) | 15 (36.59) | 17 (39.53) |  |
| ***Probiotics*** |  |  |  |  |
| No | 56 (66.67) | 22 (53.66) | 34 (79.07) | **0.01** |
| Yes | 28 (33.33) | 19 (46.34) | 9 (20.93) |  |
| ***Intravenous rehydration*** |  |  |  |  |
| No | 78 (92.86) | 37 (90.24) | 41 (95.35) | 0.43 |
| Yes | 6 (7.14) | 4 (9.76) | 2 (4.65) |  |
| ***Antibiotics or antivirus*** |  |  |  |  |
| No | 76 (90.48) | 36 (87.80) | 40 (93.02) | 0.48 |
| Yes | 8 (9.52) | 5 (12.20) | 3 (6.98) |  |
| ***Chinese patent medicine*** |  |  |  |  |
| No | 63 (75.00) | 30 (73.17) | 33 (76.74) | 0.71 |
| Yes | 21 (25.00) | 11 (26.83) | 10 (23.26) |  |
| ***Fever and pain reliever*** |  |  |  |  |
| No | 74 (88.10) | 35 (85.37) | 39 (90.70) | 0.52 |
| Yes | 10 (11.90) | 6 (14.63) | 4 (9.30) |  |
| ***Others*** |  |  |  |  |
| No | 48 (57.14) | 21 (51.22) | 27 (62.79) | 0.28 |
| Yes | 36 (42.86) | 20 (48.78) | 16 (37.21) |  |
| ***Numbers of the above Treatment/medications (mean[SD])*** | 1.8 (1.2) | 2.0 (1.3) | 1.5 (1.0) | **0.03** |
| SD: standard deviation |  |  |  |  |
| P-values based on T-test for continuous variable and Chi-square test or Fisher’s exact test (when expected cell counts less than 5) for binary or categorical variable | | | | |

| **Supplementary Table 5. Usual care from Day 1 to Day 3 among the participating children (N=84)** | | | | |
| --- | --- | --- | --- | --- |
| **Treatment** | **All** | **Sham Tuina (N=41)** | **Pediatric Tuina(N=43)** | **P value** |
| **Day 1** |  |  |  |  |
| **Oral rehydration (N[%])** |  |  |  |  |
| No | 52 (61.90) | 24 (58.54) | 28 (65.12) | 0.54 |
| Yes | 32 (38.10) | 17 (41.46) | 15 (34.88) |  |
| **Mucosal Protective Agents (N[%])** |  |  |  |  |
| No | 37 (44.05) | 18 (43.90) | 19 (44.19) | 0.98 |
| Yes | 47 (55.95) | 23 (56.10) | 24 (55.81) |  |
| **Probiotics (N[%])** |  |  |  |  |
| No | 37 (44.05) | 19 (46.34) | 18 (41.86) | 0.68 |
| Yes | 47 (55.95) | 22 (53.66) | 25 (58.14) |  |
| **Intravenous rehydration (N[%])** |  |  |  |  |
| No | 60 (71.43) | 32 (78.05) | 28 (65.12) | 0.19 |
| Yes | 24 (28.57) | 9 (21.95) | 15 (34.88) |  |
| **Antibiotics or antivirus (N[%])** |  |  |  |  |
| No | 67 (79.76) | 30 (73.17) | 37 (86.05) | 0.14 |
| Yes | 17 (20.24) | 11 (26.83) | 6 (13.95) |  |
| **Chinese patent medicine (N[%])** |  |  |  |  |
| No | 41 (48.81) | 16 (39.02) | 25 (58.14) | 0.08 |
| Yes | 43 (51.19) | 25 (60.98) | 18 (41.86) |  |
| **Fever and pain reliever (N[%])** |  |  |  |  |
| No | 78 (92.86) | 39 (95.12) | 39 (90.70) | 0.68 |
| Yes | 6 (7.14) | 2 (4.88) | 4 (9.30) |  |
| **Others (N[%])** |  |  |  |  |
| No | 54 (64.29) | 30 (73.17) | 24 (55.81) | 0.10 |
| Yes | 30 (35.71) | 11 (26.83) | 19 (44.19) |  |
| **Numbers of the above usual care (mean[SD])** | 2.9 (1.2) | 2.9 (1.1) | 2.9 (1.4) | 0.99 |
| **Day 2** |  |  |  |  |
| **Oral rehydration (N[%])** |  |  |  |  |
| No | 59 (70.24) | 26 (63.41) | 33 (76.74) | 0.18 |
| Yes | 25 (29.76) | 15 (36.59) | 10 (23.26) |  |
| **Mucosal Protective Agents (N[%])** |  |  |  |  |
| No | 37 (44.05) | 17 (41.46) | 20 (46.51) | 0.64 |
| Yes | 47 (55.95) | 24 (58.54) | 23 (53.49) |  |
| **Probiotics (N[%])** |  |  |  |  |
| No | 38 (45.24) | 18 (43.90) | 20 (46.51) | 0.81 |
| Yes | 46 (54.76) | 23 (56.10) | 23 (53.49) |  |
| **Intravenous rehydration (N[%])** |  |  |  |  |
| No | 66 (78.57) | 32 (78.05) | 34 (79.07) | 0.91 |
| Yes | 18 (21.43) | 9 (21.95) | 9 (20.93) |  |
| **Antibiotics or antivirus (N[%])** |  |  |  |  |
| No | 67 (79.76) | 31 (75.61) | 36 (83.72) | 0.36 |
| Yes | 17 (20.24) | 10 (24.39) | 7 (16.28) |  |
| **Chinese patent medicine (N[%])** |  |  |  |  |
| No | 51 (60.71) | 20 (48.78) | 31 (72.09) | **0.03** |
| Yes | 33 (39.29) | 21 (51.22) | 12 (27.91) |  |
| **Fever and pain reliever (N[%])** |  |  |  |  |
| No | 82 (97.62) | 40 (97.56) | 42 (97.67) | 1.00 |
| Yes | 2 (2.38) | 1 (2.44) | 1 (2.33) |  |
| **Others (N[%])** |  |  |  |  |
| No | 58 (69.05) | 27 (65.85) | 31 (72.09) | 0.54 |
| Yes | 26 (30.95) | 14 (34.15) | 12 (27.91) |  |
| **Numbers of the above usual care (mean[SD])** | 2.5 (1.4) | 2.9 (1.2) | 2.3 (1.5) | **0.047** |
| **Day 3** |  |  |  |  |
| **Oral rehydration (N[%])** |  |  |  |  |
| No | 64 (76.19) | 31 (75.61) | 33 (76.74) | 0.90 |
| Yes | 20 (23.81) | 10 (24.39) | 10 (23.26) |  |
| **Mucosal Protective Agents (N[%])** |  |  |  |  |
| No | 41 (48.81) | 22 (53.66) | 19 (44.19) | 0.39 |
| Yes | 43 (51.19) | 19 (46.34) | 24 (55.81) |  |
| **Probiotics (N[%])** |  |  |  |  |
| No | 39 (46.43) | 20 (48.78) | 19 (44.19) | 0.67 |
| Yes | 45 (53.57) | 21 (51.22) | 24 (55.81) |  |
| **Intravenous rehydration (N[%])** |  |  |  |  |
| No | 67 (79.76) | 33 (80.49) | 34 (79.07) | 0.87 |
| Yes | 17 (20.24) | 8 (19.51) | 9 (20.93) |  |
| **Antibiotics or antivirus (N[%])** |  |  |  |  |
| No | 66 (78.57) | 32 (78.05) | 34 (79.07) | 0.91 |
| Yes | 18 (21.43) | 9 (21.95) | 9 (20.93) |  |
| **Chinese patent medicine (N[%])** |  |  |  |  |
| No | 55 (65.48) | 23 (56.10) | 32 (74.42) | 0.08 |
| Yes | 29 (34.52) | 18 (43.90) | 11 (25.58) |  |
| **Fever and pain reliever (N[%])** |  |  |  |  |
| No | 83 (98.81) | 41 (100.00) | 42 (97.67) | 1.00 |
| Yes | 1 (1.19) | 0 (0.00) | 1 (2.33) |  |
| **Others (N[%])** |  |  |  |  |
| No | 61 (72.62) | 29 (70.73) | 32 (74.42) | 0.81 |
| Yes | 23 (27.38) | 12 (29.27) | 11 (25.58) |  |
| **Numbers of the above usual care (mean[SD])** | 2.3 (1.4) | 2.4 (1.3) | 2.3 (1.6) | 0.84 |
| **Numbers of the above usual care from Day 1 to Day 2 (mean[SD])** | 3.3 (1.2) | 3.4 (1.2) | 3.3 (1.3) | 0.50 |
| **Numbers of the above usual care from Day 1 to Day 3 (mean[SD])** | 3.5 (1.3) | 3.5 (1.1) | 3.5 (1.4) | 1.00 |
| SD: standard deviation |  |  |  |  |
| P-values based on T-test for continuous variable and Chi-square test or Fisher’s exact test (when expected cell counts less than 5) for binary variable | | | | |

| **Supplementary Table 6. Concomitant treatment from Day 1 to Day 3 among the participating children (N=84)** | | | | |
| --- | --- | --- | --- | --- |
| **Concomitant treatment** | **All** | **Sham Tuina (N=41)** | **Pediatric Tuina (N=43)** | **P value** |
| **Day 1** |  |  |  |  |
| No | 75 (89.29) | 36 (87.80) | 39 (90.70) | 0.74 |
| Yes | 9 (10.71) | 5 (12.20) | 4 (9.30) |  |
| **Day 2** |  |  |  |  |
| No | 74 (88.10) | 34 (82.93) | 40 (93.02) | 0.19 |
| Yes | 10 (11.90) | 7 (17.07) | 3 (6.98) |  |
| **Day 3** |  |  |  |  |
| No | 75 (89.29) | 34 (82.93) | 41 (95.35) | 0.09 |
| Yes | 9 (10.71) | 7 (17.07) | 2 (4.65) |  |
| **From Day 1 to Day 2** |  |  |  |  |
| No | 73 (86.90) | 34 (82.93) | 39 (90.70) | 0.35 |
| Yes | 11 (13.10) | 7 (17.07) | 4 (9.30) |  |
| **From Day 1 to Day3** |  |  |  |  |
| No | 73 (86.90) | 34 (82.93) | 39 (90.70) | 0.35 |
| Yes | 11 (13.10) | 7 (17.07) | 4 (9.30) |  |
| P-values based on Chi-square test or Fisher’s exact test (when expected cell counts less than 5). | | | | |

| **Supplementary Table 7. Association between intervention and outcomes among the children younger than 2 years (N=63, Intention-to-treat analysis)** | | | |
| --- | --- | --- | --- |
| **Outcome** | **All** | **Sham Tuina (N=32)** | **Pediatric Tuina (N=31)** |
| **Times of diarrhea on day 3** |  |  |  |
| Mean (SD) | 5.4 (4.3) | 6.0 (5.0) | 4.6 (3.3) |
| Median (IQR) | 4 (2,7) | 5.5 (3,8) | 4 (2,6) |
| Crude RR (95%CI) |  | Ref | **0.77 (0.61-0.97)** |
| Adjusted RR (95%CI)a |  | Ref | **0.71 (0.53-0.96)** |
| **Days of diarrhea from baseline** |  |  |  |
| Mean (SD) | 4.8 (3.5) | 4.7 (3.3) | 5.0 (3.6) |
| Median (IQR) | 4 (3,6) | 4 (3,6) | 4 (3,6) |
| Crude RR (95%CI) |  | Ref | 1.07 (0.84-1.36) |
| Adjusted RR (95%CI)b |  | Ref | 0.99 (0.76-1.29) |
| **Evaluation at Day 7** |  |  |  |
| Much better (N[%]) | 40 (63.5) | 23 (71.9) | 17 (54.8) |
| Slightly better or no change (ref) (N[%]) | 15 (23.8) | 6 (18.8) | 9 (29.0) |
| Missing (N[%]) | 8 (12.7) | 3 (9.4) | 5 (16.1) |
| Crude OR (95%CI) |  | Ref | 0.49 (0.15-1.65) |
| Adjusted OR (95%CI)b |  | Ref | 0.66 (0.17-2.65) |
| **Evaluation at Day 14** |  |  |  |
| Much better (N[%]) | 41 (65.1) | 22 (68.8) | 19 (61.3) |
| Slightly better or no change (ref) (N[%]) | 13 (20.6) | 7 (21.9) | 6 (19.4) |
| Missing (N[%]) | 9 (14.3) | 3 (9.4) | 6 (19.4) |
| Crude OR (95%CI) |  | Ref | 1.01 (0.29-3.52) |
| Adjusted OR (95%CI)b |  | Ref | 1.63 (0.25-10.85) |
| **Number of days when the stool characteristics returned to normal** |  |  |  |
| Mean (SD) | 6.7 (3.7) | 7.1 (3.8) | 6.3 (3.6) |
| Median (IQR) | 7 (3,7) | 7 (4,8) | 7 (3,7) |
| Crude RR (95%CI) |  | Ref | 0.89 (0.72-1.08) |
| Adjusted RR (95%CI)b |  | Ref | 0.87 (0.70-1.07) |
| SD: standard deviation. IQR: interquartile range | | | |
| Risk ratios were estimated from Poisson Regression. | | | |
| Odds ratios were estimated from Logistic Regression | | | |
| ^a^ Model adjusted for age, sex, history of infectious and digestive diseases, food allergy, hours of diarrhea before randomization, numbers of treatment/medications before randomization, numbers of the symptoms before randomization, times of diarrhea during the past 24 hours before randomization, times of diarrhea on Day 2, numbers of usual care from Day 1 to Day 2, and concomitant treatment from Day 1 to Day 2. | | | |
| ^b^ Model adjusted for age, sex, history of infectious and digestive diseases, food allergy, hours of diarrhea before randomization, numbers of treatment/medications before randomization, numbers of the symptoms before randomization, numbers of usual care from Day 1 to Day 3, and concomitant treatment from Day 1 to Day 3. | | | |

| **Supplementary Table 8. Association between intervention and outcomes among the children younger than 2 years (N=49, Per-protocol analysis)** | | | |
| --- | --- | --- | --- |
| **Outcome** | **All** | **Sham Tuina (N=28)** | **Pediatric Tuina (N=21)** |
| **Times of diarrhea on day 3** |  |  |  |
| Mean (SD) | 5.6 (4.5) | 6.4 (5.0) | 4.4 (3.6) |
| Median (IQR) | 4 (2,8) | 6 (3,8.5) | 4 (2,6) |
| Crude RR (95%CI) |  | Ref | **0.69 (0.54-0.89)** |
| Adjusted RR (95%CI)a |  | Ref | 0.73 (0.54-1.00)c |
| **Days of diarrhea from baseline** |  |  |  |
| Mean (SD) | 5.1 (3.7) | 5 (3.3) | 5.3 (4.2) |
| Median (IQR) | 4 (3,6.5) | 4 (3,6) | 4 (2,8) |
| Crude RR (95%CI) |  | Ref | 1.07 (0.82-1.39) |
| Adjusted RR (95%CI)b |  | Ref | 0.93 (0.70-1.25) |
| **Evaluation at Day 7** |  |  |  |
| Much better (N[%]) | 35 (71.4) | 21 (75.0) | 14 (66.7) |
| Slightly better or no change (ref) (N[%]) | 13 (26.5) | 6 (21.4) | 7 (33.3) |
| Missing (N[%]) | 1 (2.0) | 1 (3.6) | 0 (0.0) |
| Crude OR (95%CI) |  | Ref | 0.57 (0.16-2.06) |
| Adjusted OR (95%CI)b |  | Ref | 0.64 (0.14-2.99) |
| **Evaluation at Day 14** |  |  |  |
| Much better (N[%]) | 35 (71.4) | 20 (71.4) | 15 (71.4) |
| Slightly better or no change (ref) (N[%]) | 11 (22.4) | 7 (25.0) | 4 (19.0) |
| Missing (N[%]) | 3 (6.1) | 1 (3.6) | 2 (9.5) |
| Crude OR (95%CI) |  | Ref | 1.31 (0.32-5.32) |
| Adjusted OR (95%CI)b |  | Ref | 1.75 (0.20-15.26) |
| **Number of days when the stool characteristics returned to normal** |  |  |  |
| Mean (SD) | 7.0 (3.8) | 7.4 (3.7) | 6.6 (3.8) |
| Median (IQR) | 7 (4,8) | 7 (4,9) | 7 (3,7) |
| Crude RR (95%CI) |  | Ref | 0.90 (0.73-1.12) |
| Adjusted RR (95%CI)b |  | Ref | 0.86 (0.68-1.08) |
| SD: standard deviation. IQR: interquartile range | | | |
| Risk ratios were estimated from Poisson Regression. | | | |
| Odds ratios were estimated from Logistic Regression | | | |
| ^a^ Model adjusted for age, sex, history of infectious and digestive diseases, food allergy, hours of diarrhea before randomization, numbers of treatment/medications before randomization, numbers of the symptoms before randomization, times of diarrhea during the past 24 hours before randomization, times of diarrhea on Day 2, numbers of usual care from Day 1 to Day 2, and concomitant treatment from Day 1 to Day 2. | | | |
| ^b^ Model adjusted for age, sex, history of infectious and digestive diseases, food allergy, hours of diarrhea before randomization, numbers of treatment/medications before randomization, numbers of the symptoms before randomization, numbers of usual care from Day 1 to Day 3, and concomitant treatment from Day 1 to Day 3. | | | |
| ^c^ P=0.052 | | | |
